# Intensivist coverage and critically ill COVID-19 patient outcomes: a population-based cohort study

**DOI:** 10.1186/s40560-023-00668-1

**Published:** 2023-05-12

**Authors:** Tak Kyu Oh, Saeyeon Kim, In-Ae Song

**Affiliations:** 1 Department of Anesthesiology and Pain Medicine, Seoul National University Bundang Hospital, Gumi-Ro, 173, Beon-Gil, Bundang-Gu, Seongnam, 13620 South Korea; 2 Department of Anesthesiology and Pain Medicine, College of Medicine, Seoul National University, Seoul, South Korea; 3 Interdepartment of Critical Care Medicine, Seoul National University Bundang Hospital, Seongnam, South Korea

**Keywords:** In-hospital mortality, Intensive care unit, Intensivists

## Abstract

**Background:**

Trained intensivist staffing improves survival outcomes in critically ill patients at intensive care units. However, the impact on outcomes of critically ill patients with coronavirus disease 2019 has not yet been evaluated. We aimed to investigate whether trained intensivists affect outcomes among critically ill coronavirus disease 2019 patients in South Korean intensive care units.

**Methods:**

Using a nationwide registration database in South Korea, we included adult patients admitted to the intensive care unit from October 8, 2020, to December 31, 2021, with a main diagnosis of coronavirus disease 2019. Critically ill patients admitted to intensive care units that employed trained intensivists were included in the intensivist group, whereas all other critically ill patients were assigned to the non-intensivist group.

**Results:**

A total of 13,103 critically ill patients were included, with 2653 (20.2%) patients in the intensivist group and 10,450 (79.8%) patients in the non-intensivist group. In the covariate-adjusted multivariable logistic regression model, the intensivist group exhibited 28% lower in-hospital mortality than that of the non-intensivist group (odds ratio: 0.72; 95% confidence interval: 0.62, 0.83; *P* < 0.001).

**Conclusions:**

Trained intensivist coverage was associated with lower in-hospital mortality among critically ill coronavirus disease 2019 patients who required intensive care unit admission in South Korea.

**Supplementary Information:**

The online version contains supplementary material available at 10.1186/s40560-023-00668-1.

## Background

Coronavirus disease 2019 (COVID-19) is a public health crisis that was declared a pandemic by the World Health Organization (WHO) on March 11, 2020 [1]. From December 8, 2020, vaccination for COVID-19 began globally, and the COVID-19 pandemic has transitioned to an endemic state [2]. The COVID-19 Excess Mortality Collaborators group reported that 18.2 million people died worldwide between January 1, 2020, and December 31, 2021 due to COVID-19 [3]. Although endemic COVID-19 infection is associated with less severe symptoms [4], many patients with COVID-19 still require admission to the intensive care unit (ICU) due to severe COVID-19 symptoms.

The staffing pattern of ICU physicians has been an issue for debate [5]. A closed ICU model in which critically ill patients were admitted under the full responsibility of a trained intensivist yielded better clinical outcomes among ICU patients than did the open ICU model in which patients were not managed by trained intensivists [5–8], probably because all procedures or decisions associated with life-sustaining treatments in the ICU are generally made at the discretion of intensivists [9]. In this context, we recently reported that intensive care in South Korea was associated with better survival outcomes in critically ill patients admitted to the South Korean ICU from 2016 to 2019 [10]. The COVID-19 pandemic has required the dedication of intensivists for the management of ICU patients, also affecting the well-being of intensivists [11]. However, there is no clear information regarding the impact of intensivist coverage on survival outcomes among critically ill COVID-19 patients in the ICU.

Therefore, using a nationwide registration database in South Korea from 2020, we aimed to investigate whether trained intensivists affected outcomes among critically ill COVID-19 patients in South Korean ICUs. We hypothesized that the presence of trained intensivists in ICUs leads to improved survival outcomes among critically ill COVID-19 patients. While our previous study from 2016 to 2019 [10] relied solely on data from the NHIS database, the present study utilized a database that was jointly created by the NHIS and the Korea Disease Control and Prevention Agency (KDCA) through a collaboration established during the COVID-19 pandemic.

## Methods

### Study design, setting, and ethical considerations

This population-based cohort study followed the Strengthening the Reporting of Observational Studies in Epidemiology guidelines [12]. The Institutional Review Board (IRB) of Seoul National University Bundang Hospital waived the requirement for IRB approval of this study because of its use of public data available to all researchers (IRB number: X-2205-758-901). The requirement for informed consent was waived by the IRB because the study was based on a retrospective analysis of anonymized data.

### KDCA-COVID19-NHIS cohort (data source)

We used data from the KDCA and National Health Insurance Service (NHIS). The KDCA-COVID19-NHIS cohort was generated for academic purposes through the collaboration of the KCDA and NHIS in South Korea. The KCDA initially extracted data regarding patients confirmed with COVID-19 by polymerase chain reaction (PCR) test from October 8, 2020, to December 31, 2021. The data from the KCDA contained information regarding age, sex, date of COVID-19 diagnosis using the PCR test, date of death, date of vaccination (1st, 2nd, and 3rd), and type of infection route. The type of infection route was classified into six groups: (1) inflow from foreign countries, (2) contact with person-related inflow from foreign countries, (3) outbreak in hospitals or nursing care centers, (4) outbreak in local communities, (5) contact with a confirmed patient, and (6) unknown. In addition to data from the KDCA, the NHIS extracted information regarding demographic and socioeconomic status, all disease diagnoses using the International Classification of Diseases (ICD)-10 codes, and prescription information of any procedures or drugs until March 31, 2022. Both KDCA and NHIS approved the data sharing for this study (research grant number: KDCA-NHIS-2022-1-489).

### Study population

This study included adult patients (≥ 18 years old) confirmed with COVID-19 by PCR test who were admitted to the ICU with a main diagnosis of COVID-19. To focus on the last episode of ICU admission in a patient, in multiple (≥ 2) admission cases the earlier ICU admissions were excluded. These multiple ICU admissions included transfer from hospital to hospital. For example, if a patient was transferred from the ICU of a general hospital to the ICU of a tertiary general hospital, only the last ICU admission at the tertiary general hospital would be included in this study.

### Trained intensivist system in South Korea

In this study, a trained intensivist was defined as an individual who was certified by the Korean Society of Critical Care Medicine after completing a fellowship training program in intensive care medicine. The fellowship program includes a compulsory one-year immersion in the ICU at the training hospital of the intensivist, an examination, an interview, the presentation of an abstract at the annual congress of the Korean Society of Critical Care Medicine, and the publication of an original article in the official publication of the society. Doctors specializing in internal medicine, anesthesiology and pain medicine, pediatrics, neurology, neurosurgery, emergency medicine, general surgery, and thoracic surgery are eligible to apply for the fellowship training course. As reported in previous studies [10, 13], South Korea implemented a special payment system for trained intensivist coverage from August 2015. This special payment system applies to medical centers that hire trained intensivists for the management of ICU patients. Trained intensivists must work only in the ICU (not in the ward) for at least ≥ 8 h/day and ≥ 5 days/week. Moreover, there should be at least one intensivist per ICU in a hospital to receive special payments.

In the present study, critically ill COVID-19 patients admitted to ICUs that hired trained intensivists were assigned to the intensivist group, whereas all other critically ill COVID-19 patients were assigned to the non-intensivist group. Critically ill COVID-19 patients who were admitted to an ICU that did not have a trained intensivist were managed by doctors other than trained intensivists.

### ICU management during the COVID-19 pandemic in South Korea

As all patients admitted to the ICU must be registered in the NHIS database using ICU prescription codes, there were no missing cases. Since the start of the COVID-19 pandemic, from 2020 to the present, the Central Disease Control Headquarters in South Korea have overseen the national-level countermeasures against infectious diseases [14]. First, general ICUs (i.e., those not dedicated to patients with COVID-19) were required to be designated as ICUs for COVID-19 patients; this decision was based on the number of critically ill patients and the increasing social isolation level in the community. Second, a system for transferring and treating critically ill patients to other regions, according to the number of COVID-19 patients in certain areas with available ICU beds, was established nationwide. Through this strategy, the Central Disease Control Headquarters attempted to ensure that there would not be a lack of ICU beds for critically ill COVID-19 patients during the pandemic. Additionally, to prevent a shortage of medical staff to care for such patients, doctors in the military, public health doctors, and resting nurses were assigned to ICUs as required.

Although ICUs were newly and rapidly constructed to take care of critically ill COVID-19 patients, they still had to meet certain legal requirements. First, emergency resuscitation devices, intubation devices, mechanical ventilators, defibrillators, electrocardiograms, and respiratory function measuring devices had to be available for use at all times. Second, they were required to include a dedicated space that was larger than the general ward, with at least 10 square meters per patient. Third, they were required to always have a dedicated doctor and at least one nurse for every two patients. Fourth, as patients may die during power outages, they needed to be equipped with uninterruptible power supply. The critically ill COVID-19 patients who were admitted to the ICU in this study included patients who were admitted to both existing and newly constructed ICUs in South Korea.

### Study outcomes

The primary outcome of this study was in-hospital mortality, defined as death during hospitalization-associated COVID-19.

### Included covariates

Age and sex were collected as demographic information. To determine the socioeconomic status of critically ill COVID-19 patients, employment status, the household income level, and residence at the time of admission due to COVID-19 infection were collected. The household income level was evaluated differently for employee-insured and self-employed insured individuals. For employee-insured individuals, the insurance premium was determined solely based on their income. On the other hand, for self-employed insured individuals, the insurance premium was determined based on their income, property, living standards, and rate of participation in economic activities. Of note, those who could not afford insurance premiums or had difficulty financially supporting themselves were included in the medical aid program instead. In addition to the medical aid program group, the household income level was classified into four quartile ratios (from Q1, the lowest, to Q4, the highest). The residences of critically ill COVID-19 patients were classified as urban (Seoul and other metropolitan cities) or rural (all other areas). Data regarding hospital level (A, B, C, and D; detailed information is presented in the statistical methodology section) and total case volume of ICU admissions due to COVID-19 during the study period were collected. All patients were classified into four groups according to the hospital where they were admitted to the ICU using quartile ratios (Q1: 0–150, Q2: 151–257, Q3: 258–408, and Q4: ≥ 409). To reflect the severity of critically ill patients with COVID-19, we utilized the WHO clinical progression scale without the P/F ratio [15]. Treatment information from the day of ICU admission or the day after ICU admission was used to indicate the initial severity of such patients. The clinical progression scale used in this study classified patients as follows: 1 point (no oxygen therapy), 2 points (oxygen by mask or nasal prongs), 3 points (oxygen by non-invasive ventilation or high flow nasal cannula [HFNC]), 4 points (intubation and mechanical ventilation), 5 points (mechanical ventilation with vasopressor use), and 6 points (mechanical ventilation with vasopressor use, dialysis, or extracorporeal membrane oxygenation [ECMO]). The vasopressors used included norepinephrine, epinephrine, dopamine, dobutamine, and vasopressin. In addition, ICD-10 coding-based acute respiratory distress syndrome (ARDS, J80) diagnosis during hospitalization due to COVID-19 was collected as a covariate. With regard to the comorbid status of patients, the Charlson comorbidity index (CCI) and disability at ICU admission were collected. CCI scores were calculated using ICD-10 codes—2020–2021 which were registered in the NHIS database (Additional file 1: Table S1). All individuals with any disabilities should be registered in the NHIS database in order to receive various social welfare benefits. Disabilities were categorized into the following 15 types: physical and brain lesion disabilities; visual disturbances; hearing and speech disabilities; autism; intellectual, mental, renal, heart, and respiratory disorders; hepatopathies; facial disfigurements; intestinal and urinary fistulae; and epilepsy. The degree of each disability was divided into the following two groups according to the severity criteria: severe disability, and mild-to-moderate disability. Disability was diagnosed and determined according to the laws of a specialty physician in each field. The most important criterion for determining disability was whether it interfered with maintaining daily life.

### Statistical methodology

The Mann–Whitney U test for continuous variables and the Chi-square test for categorical variables were used to compare the clinicopathological characteristics of patients between the intensivist and non-intensivist groups. We first selected covariates based on patient factors that might affect the prognosis of patients with COVID-19, such as age, sex, CCI, disability, and history of vaccination. We also selected covariates related to socioeconomic status factors, as socioeconomic status can have an impact on COVID-19 patients’ outcomes [16]. The type of infection route and WHO clinical progression scale were also collected because the characteristics and prognosis of COVID-19 infection may differ based on these factors [17]. Lastly, total case volume and hospital levels were collected to reflect the capacity of each hospital where critically ill patients with COVID-19 were admitted to the ICU.

Among the covariates, hospital levels for the critically ill COVID-19 patients were identified using a hierarchical approach. For hierarchical cluster analysis, agglomerative clustering was performed using hospital-related variables, such as the type of hospital (general hospital or long-term care facility), total number of doctors, specialist doctors, nurses, pharmacists, hospital beds, operating room beds, adult ICU beds, and emergency room beds. Four groups were created based on the results of the hierarchical clustering analysis; the characteristics of the four hospital groups are presented in Additional file 2: Table S2.

We constructed a multivariate logistic regression model for in-hospital mortality among critically ill COVID-19 patients. The selected covariates were included in the model for multivariable adjustment, and the results are presented as odds ratios (OR) with 95% confidence intervals (CI). We also performed subgroup analyses according to the WHO clinical progression scale, ARDS diagnosis, and hospital level. Hosmer–Lemeshow statistics were used to confirm that the goodness of fit in the multivariable model was appropriate. There was no issue regarding multicollinearity within the variables with the criterion of variance inflation factors < 2.0. All statistical analyses were performed using IBM SPSS Statistics for Windows (version 25.0; IBM Corp., Armonk, NY, USA). A *P*-value of < 0.05 was considered statistically significant.

## Results

### Study population

**Fig. 1:**
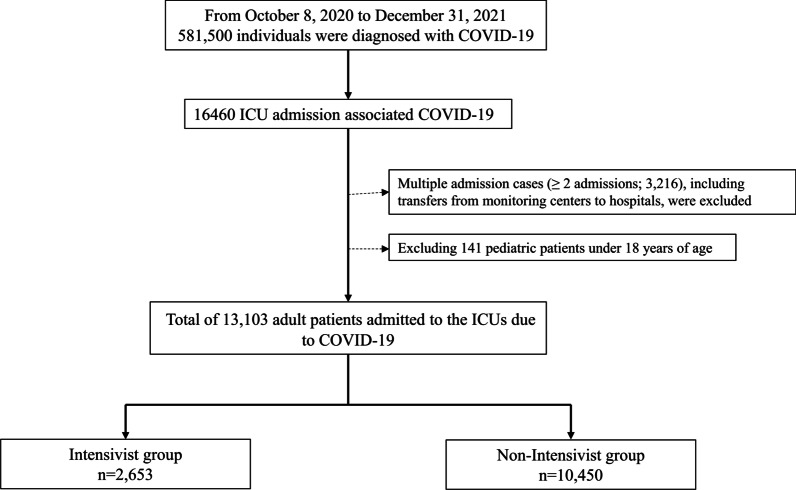
Flow chart depicting the selection process of critically ill COVID-19 patients

From October 8, 2020, to December 31, 2021, 581,500 patients were confirmed with COVID-19 by PCR test and admitted to hospitals or government-managed monitoring centers. Among them, there were 16,460 ICU admissions due to COVID-19. After excluding 3216 multiple cases of ICU admission due to COVID-19 and 141 pediatric patients, a total of 13,103 adult patients admitted to the ICUs due to COVID-19 were included. A total of 2653 (20.2%) patients were admitted to ICUs covered by a trained intensivist, whereas 10,450 (79.8%) patients were admitted to ICUs that were not covered by a trained intensivist, as shown in Fig. 1. Table 1 shows a comparison of the clinicopathological characteristics between the intensivist and non-intensivist groups. The median value of age was higher in the intensivist group (67 years [interquartile range, IQR: 52, 75]) than in the non-intensivist group (65 years [IQR: 51, 74]), while the proportion of men was higher in the intensivist group (60.6%, 1607/2653) than in the non-intensivist group (55.6%, 5808/10,450).

**Table 1:** Comparison of the clinicopathological characteristics between the intensivist and non-intensivist groups

Variable	Intensivist group n = 2653	Non-intensivist group n = 10,450	*P*-value
Age, year	67 [52, 75]	65 [51, 74]	< 0.001
Male sex	1607 (60.6)	5808 (55.6)	< 0.001
Having a job	1460 (55.0)	5813 (55.6)	0.582
Household income level			0.190
Medical aid group	253 (9.5)	964 (9.2)	
Q1 (lowest)	488 (18.4)	1869 (17.9)	
Q2	457 (17.2)	1874 (17.9)	
Q3	523 (19.7)	2256 (21.6)	
Q4 (highest)	907 (34.2)	3373 (32.3)	
Unknown	25 (0.9)	114 (1.1)	
Residence			< 0.001
Urban area	1519 (57.3)	5355 (51.2)	
Rural area	1134 (42.7)	5095 (48.8)	
Type of infection route			< 0.001
Inflow from foreign countries	36 (1.4)	135 (1.3)	
Contact with person-related inflow from foreign countries	1 (0.0)	4 (0.0)	
Outbreak in hospitals or nursing care centers	311 (11.7)	1581 (15.1)	
Outbreak in local communities	322 (12.1)	1216 (11.6)	
Contact with a patient confirmed with patients	722 (27.2)	3163 (30.3)	
Unknown	1,261 (47.5)	4351(41.6)	
CCI, point	5.5 [3.4, 6.4]	5.8 [3.8, 6.6]	0.020
Underlying disability			0.166
Severe	208 (7.8)	941 (9.0)	
Mild to moderate	284 (10.7)	1099 (10.5)	
Hospital level group			< 0.001
A	312 (11.8)	4,158 (39.8)	
B	185 (7.0)	529 (5.1)	
C	2,072 (78.1)	5,137 (49.2)	
D	84 (3.2)	626 (6.0)	
Total case volume of ICU admission due to COVID-19			< 0.001
Q1:0–150	1115 (42.0)	2221 (21.3)	
Q2:151–257	660 (24.9)	2632 (25.2)	
Q3:258–408	657 (24.8)	2687 (25.7)	
Q4: ≥ 409	221 (8.3)	2910 (27.8)	
1st vaccination	1559 (58.8)	6824 (65.3)	< 0.001
2nd vaccination	1378 (51.9)	6271 (60.0)	< 0.001
3rd vaccination	692 (26.1)	3485 (33.3)	< 0.001
WHO clinical progression scale			
1 point (no oxygen therapy)	451 (17.1)	1672 (16.0)	0.215
2 points (oxygen by mask or nasal prongs)	944 (35.6)	3887 (37.2)	< 0.001
3 points (oxygen by NIV or HFNC)	565 (21.3)	1818 (17.4)	< 0.001
4 points (intubation and MV)	384 (14.5)	1285 (12.3)	< 0.001
5 points (MV with vasopressor use)	208 (7.8)	1642 (15.7)	< 0.001
6 points (MV and vasopressor use, dialysis or ECMO)	101 (3.8)	146 (1.4)	< 0.001
Diagnosis of ARDS	326 (12.3)	741 (7.1)	< 0.001
In-hospital mortality	715 (27.0)	1990 (19.0)	< 0.001

Median value with interquartile range was used for continuous variable and number with percentage was used for categorical variable

COVID-19, Coronavirus disease-2019; CCI, Charlson comorbidity index; LOS, length of hospital stays; ICU, intensive care unit; WHO, world health organization; NIV. Noninvasive ventilation; HFNC, high flow nasal cannula; MV, mechanical ventilation; ECMO, extracorporeal membrane oxygenation; ARDS, acute respiratory distress syndrome

### In-hospital mortality

Table 2 shows the results of uni- and multivariable logistic regression analyses for in-hospital mortality among critically ill COVID-19 patients. The univariable logistic regression analysis revealed no significant difference for in-hospital mortality between the intensivist and non-intensivist groups (OR: 0.96, 95% CI: 0.74, 1.25; *P* = 0.754). However, in the covariate-adjusted multivariable logistic regression model, the intensivist group exhibited 28% lower in-hospital mortality compared to the non-intensivist group (OR: 0.72, 95% CI: 0.62, 0.83; *P* < 0.001). All ORs with 95% CIs of other covariates in the multivariable model are presented in Additional file 3: Table S3. Among the analyzed covariates, compared to a score of 1 on the WHO clinical progression scale, a score of 2 (OR: 1.06, 95% CI: 1.06, 1.07; *P* < 0.001), 3 (OR: 1.12, 95% CI: 1.06, 1.19; *P* < 0.001), 4 (OR: 2.45, 95% CI: 1.76, 3.43; *P* < 0.001), 5 (OR: 3.83, 95% CI: 2.59, 5.65; *P* < 0.001), and 6 (OR: 12.23, 95% CI: 10.50, 15.32; *P* < 0.001) were associated with higher in-hospital mortality. Moreover, the diagnosis of ARDS during hospitalization was associated with 60% (OR: 1.60, 95% CI: 1.38, 2.00; *P* < 0.001) higher in-hospital mortality rate compared to that of critically ill COVID-19 patients who were not diagnosed with ARDS.

**Table 2:** Uni- and multivariable logistic regression analyses for in-hospital mortality among critically ill COVID-19 patients

Variable	OR (95% CI)	*P*-value
Unadjusted		
Intensivist group (vs non-intensivist group)	0.96 (0.74, 1.25)	0.754
Covariate-adjusted		
Intensivist group (vs non-intensivist group)	0.72 (0.62, 0.83)	< 0.001

OR, odds ratio; CI, confidence interval

### Subgroup analyses

Table 3 shows the results of the subgroup analysis for in-hospital mortality. The intensivist group showed significantly lower in-hospital mortality than did the non-intensivist group among patients with 2 points (OR: 0.75, 95% CI: 0.60, 0.89; *P* < 0.002), 3 points (OR: 0.73, 95% CI: 0.60, 0.87; *P* < 0.001), 4 points (OR: 0.67, 95% CI: 0.45, 0.82; *P* < 0.001), and 6 points (OR: 0.68, 95% CI: 0.47, 0.99; *P* = 0.048) on the WHO clinical progression scale. Among patients who were diagnosed with ARDS, the intensivist group showed lower in-hospital mortality compared to the non-intensivist group (OR: 0.64; 95% CI: 0.41, 0.98; *P* = 0.042).

**Table 3:** Subgroup analysis for in-hospital mortality

Variable	OR (95% CI)	*P*-value
WHO clinical progression scale: 1 point		
Intensivist group (vs non-intensivist group)	0.80 (0.55, 1.28)	0.375
WHO clinical progression scale: 2 points		
Intensivist group (vs non-intensivist group)	0.75 (0.60, 0.89)	< 0.001
WHO clinical progression scale: 3 points		
Intensivist group (vs non-intensivist group)	0.73 (0.60, 0.87)	< 0.001
WHO clinical progression scale: 4 points		
Intensivist group (vs non-intensivist group)	0.67 (0.45, 0.82)	< 0.001
WHO clinical progression scale: 5 points		
Intensivist group (vs non-intensivist group)	0.92 (0.80, 1.18)	0.218
WHO clinical progression scale: 6 points		
Intensivist group (vs non-intensivist group)	0.68 (0.47, 0.99)	0.048
ARDS group		
Intensivist group (vs non-intensivist group)	0.64 (0.41, 0.98)	0.042
Hospital level group A		
Intensivist group (vs non-intensivist group)	0.89 (0.59, 1.34)	0.577
Hospital level group B		
Intensivist group (vs non-intensivist group)	0.60 (0.28, 1.25)	0.170
Hospital level group C		
Intensivist group (vs non-intensivist group)	0.72 (0.59, 0.88)	0.001
Hospital level group D		
Intensivist group (vs non-intensivist group)	0.55 (0.23, 1.35)	0.191

*OR* odds ratio, *CI* confidence interval, *HFNC* high flow nasal cannula, *ECMO* extracorporeal membrane oxygenation, *CRRT* continuous renal replacement therapy, *ARDS* acute respiratory distress syndrome

## Discussion

In this population-based cohort study conducted in South Korea, trained intensivist coverage was associated with improved survival outcomes among critically ill COVID-19 patients. This association was significant in critically ill COVID-19 patients who were diagnosed with ARDS or received therapy, such as nasal or mask oxygen therapy, HFNC therapy, mechanical ventilatory support, or ECMO support. Our results suggest that it is important to employ a large number of trained intensivists in ICUs to manage public infection crises, such as COVID-19.

Similar to other common viral pneumonias, care for critically ill patients with COVID-19 has been a critical issue globally [18]. ICUs require expensive facilities and many experienced medical staff for critically ill patients, and the scarcity of ICU resources during the COVID-19 pandemic was a global issue [19]. Therefore, devising a strategy for the allocation of ventilator and ICU resources was a challenging issue [20]. In addition to the issues related to ICU resources, the lack of human resources, such as trained doctors and experienced nurses, was an equally important problem. The COVID-19 pandemic required the redeployment of junior doctors who were not adequately trained for ICU management [21]. In this context, the beneficial impact of trained intensivists on the outcomes of critically ill COVID-19 patients remains a critical issue both at present and in the future, considering the possibility of repeated pandemics such as COVID-19, influenza, and Middle East Respiratory Syndrome.

Previous studies reported that patients who received mechanical ventilation could benefit from the care of trained intensivists [22, 23]. Moreover, ARDS is a challenging condition for intensivists due to poor disease prognosis and high mortality [24]. Importantly, the disease progression of ARDS associated with COVID-19 is not similar to that of standard ARDS, and the role of well-trained practitioners such as intensivists is important to modify and refine the management of ARDS associated with COVID-19 [25]. Many critically ill COVID-19 patients require ECMO support due to ARDS or severe hypoxemia [26], and previous studies revealed that intensivist-led teams yielded benefits in patients who received ECMO support [27, 28]. Similarly, the results of our subgroup analyses presented in Table 3 show that there were beneficial associations of intensivist coverage in critically ill COVID-19 patients who received HFNC therapy, mechanical ventilatory support, and ECMO support, in addition to patients who were diagnosed with ARDS associated with COVID-19.

Previous studies have reported the important role of intensivists for critically ill COVID-19 patients in various aspects, in addition to respiratory failure or ARDS associated with COVID-19. Previous studies reported that COVID-19 associated acute kidney injury (AKI) or cardiac injury is an important and challenging issue for intensivists in the ICU [29, 30]. This suggests that not only ARDS or respiratory failure-associated COVID-19 but also other conditions associated with COVID-19 such as AKI, cardiac injury, or sepsis could be critical issues that require intensive care by trained intensivists. In the United States, it was recently reported that the demand for intensivists dramatically increased during the COVID-19 pandemic which is now endemic; therefore, more intensivists should be trained for the future [31]. In South Korea, the impact of intensivists on outcomes among critically ill patients has been continuously reported. Lee et al. reported that the presence of intensivist staffing in the ICU was associated with a lower mortality risk during the 2011–2015 cohort in the Korean NHIS [32]. Recently, using the NHIS ICU cohort from 2016 to 2019, we reported that intensive care coverage by trained intensivists was associated with better survival outcomes in critically ill patients admitted to South Korean ICUs [10].

This study has several limitations. First, given that we collected information such as ARDS diagnosis and WHO clinical progression scale scores using treatment information on the day of ICU admission or the day after ICU admission, we could not use laboratory results due to the lack of this information in the NHIS database. Therefore, we could not evaluate the P/F ratio or Acute Physiology and Chronic Health Evaluation II scores for assessing the disease severity of critically ill COVID-19 patients. Second, we did not consider the types of COVID-19, also because of the lack of this information in the database. Various types of COVID-19, caused by omicron, delta, and alpha variants, have different clinical severities and responses to vaccination [33, 34]. Third, this study did not consider the specific working patterns of intensivists. For instance, in the South Korean specific payment system, trained intensivists are required to work a minimum of 8 h/day and 5 days/week only in the ICU. We could not capture cases where intensivists worked night shifts, weekends, or holidays. Lastly, special circumstances arising from the COVID-19 pandemic should be considered when interpretating our findings. Although the South Korean government attempted to ensure that there were no problems with the allocation of ICU beds and medical staff during the pandemic, some critically ill COVID-19 patients died while waiting for interhospital transfer or hospitalization at home. Moreover, the temporary ICU supply of medical staff during the pandemic aimed at treating COVID-19 patients may have affected our results, as the quality of care provided by temporarily supplied medical staff may have differed from that provided by regular ICU staff. However, despite these limitations, our results highlight the importance of having a sufficient number of trained intensivists during the COVID-19 pandemic for improving patient outcomes.

## Conclusions

In conclusion, trained intensivist coverage was associated with lower in-hospital mortality among critically ill COVID-19 patients who required ICU admission in South Korea.

## Supplementary Information

**Additional file 1: Table S1.** The ICD-10 codes used by comorbidity to compute the Charlson comorbidity index.

**Additional file 2: Table S2.** Characteristics of the four hospital groups.

**Additional file 3: Table S3.** All ORs with 95% CIs of other covariates in the multivariable model.

## Data Availability

All data are available upon reasonable request from the corresponding author.

## Abbreviations

ARDS: Acute respiratory distress syndrome
CCI: Charlson comorbidity index
CI: Confidence interval
COVID-19: Coronavirus disease 2019
ECMO: Extracorporeal membrane oxygenation
HFNC: High flow nasal cannula
ICD: International Classification of Diseases
ICU: Intensive care unit
IQR: Interquartile range
IRB: Institutional Review Board
KDCA: Korea Disease Control and Prevention Agency
NHIS: National Health Insurance Service
OR: Odds ratio
PCR: Polymerase chain reaction
WHO: World Health Organization
