# Supplementary material for: Intensivist coverage and critically ill COVID-19 patient outcomes: a population-based cohort study
Source: J Intensive Care. 2023 May 12;11:19. doi: 10.1186/s40560-023-00668-1 (PMC10177723; doi:10.1186/s40560-023-00668-1)
Supplement: Supplementary file 2 — Additional file 2: Table S2. Characteristics of the four hospital groups. [file 40560_2023_668_MOESM2_ESM.docx]

Table S2. Characteristics of the four hospital groups

| Hospital level | | Hospital level A  n=450 | Hospital level B  n=63 | Hospital level C  n=542 | Hospital level D  n=12 |
| --- | --- | --- | --- | --- | --- |
| Type of hospital | |  |  |  |  |
|  | General hospital | 0 (0.0) | 63 (100.0) | 432 (79.7) | 12 (100.0) |
|  | Long-term facility care center | 450 (100.0) | 0 (0.0) | 110 (20.3) | 0 (0.0) |
| Total number of doctor | | 150.9 (118.8) | 1,942.2 (373.2) | 618.8 (198.8) | 2,270.6 (318.2) |
| Total number of specialist doctor | | 66.1 (46.5) | 695.1 (116.6) | 230.9 (73.2) | 911.4 (83.6) |
| Total number of nurse | | 290.2 (210.4) | 2,587.8 (486.2) | 1,111.6 (329.9) | 3,969.3 (378.2) |
| Total number of pharmacist | | 7.7 (7.6) | 129.5 (24.5) | 31.9 (14.3) | 139.3 (23.0) |
| Total number of hospital bed | | 323.6 (136.2) | 1,547.6 (243.0) | 835.5 (185.0) | 2,344.1 (143.6) |
| Total number of OR bed | | 6.9 (3.6) | 45.6 (9.2) | 19.8 (6.2) | 72.0 (3.0) |
| Total number of adult ICU bed | | 20.1 (11.0) | 62.2 (16.7) | 51.0 (20.4) | 75.5 (0.5) |
| Total number of ER bed | | 20.4 (6.0) | 46.3 (9.9) | 31.4 (6.6) | 78.8 (28.5) |

OR, operating room; ICU, intensive care unit; ER, emergency room
