# Supplementary material for: Intensivist coverage and critically ill COVID-19 patient outcomes: a population-based cohort study
Source: J Intensive Care. 2023 May 12;11:19. doi: 10.1186/s40560-023-00668-1 (PMC10177723; doi:10.1186/s40560-023-00668-1)
Supplement: Supplementary file 3 — Additional file 3: Table S3. All ORs with 95% CIs of other covariates in the multivariable model. [file 40560_2023_668_MOESM3_ESM.docx]

Table S3. All ORs with 95% CIs of other covariates in the multivariable model

| Variable | | | OR (95% CI) | *P*-value |
| --- | --- | --- | --- | --- |
| Age | | | 1.08 (1.08, 1.09) | <0.001 |
| Female sex | | | 0.83 (0.71, 0.92) | <0.001 |
| Having a job | | | 0.97 (0.86, 1.12) | 0.602 |
| Household income level | | |  |  |
|  | Medical aid group | | 1.10 (0.87, 1.38) | 0.325 |
|  | Q1 (lowest) | | 1 |  |
|  | Q2 | | 0.80 (0.65, 0.98) | 0.039 |
|  | Q3 | | 0.98 (0.82, 1.20) | 0.637 |
|  | Q4 (highest) | | 0.84 (0.70, 0.96) | 0.020 |
|  | Unknown | | 0.80 (0.38, 1.52) | 0.421 |
| Residence | | |  |  |
|  | Urban area | | 1 |  |
|  | Rural area | | 1.20 (0.90, 1.35) | 0.218 |
| Type of infection route | | |  |  |
|  | Inflow from foreign countries or contact with person-related inflow from foreign countries | | 1 |  |
|  | Outbreak in hospitals or nursing care centers | | 1.25 (1.07, 1.48) | 0.007 |
|  | Outbreak in local communities | | 0.85 (0.68, 1.08) | 0.178 |
|  | Contact with a patient confirmed with patients | | 0.95 (0.80, 1.09) | 0.524 |
|  | Unknown | | 0.86 (0.67, 1.35) | 0.388 |
| CCI, point | | | 1.04 (1.02, 1.06) | <0.001 |
| Underlying disability | | |  |  |
|  | Severe | | 1.45 (1.15, 1.79) | <0.001 |
|  | Mild to moderate | | 1.15 (1.00, 1.38) | 0.055 |
| Hospital level group | | |  |  |
|  | A | | 1 |  |
|  | B | | 0.65 (0.47, 0.88) | <0.001 |
|  | C | | 0.58 (0.48, 0.71) | <0.001 |
|  | D | | 0.47 (0.31, 0.70) | <0.001 |
| Total case volume of COVID-19 related ICU admission | | |  |  |
|  | Q1:0–150 | | 1 |  |
|  | Q2:151–257 | | 1.18 (0.98, 1.40) | 0.102 |
|  | Q3:258–408 | | 0.86 (0.72, 1.04) | 0.082 |
|  | Q4: ≥ 409 | | 0.77 (0.60, 0.94) | 0.010 |
| 1^st^ vaccination | | | 0.59 (0.45, 0.74) | <0.001 |
| 2^nd^ vaccination | | | 0.68 (0.52, 0.87) | 0.002 |
| 3^rd^ vaccination | | | 0.10 (0.08, 0.15) | <0.001 |
| WHO clinical progression scale | | |  |  |
|  | | 1 point (no oxygen therapy) | 1 |  |
|  | | 2 points (oxygen by mask or nasal prongs) | 1.06 (1.06, 1.07) | <0.001 |
|  | | 3 points (oxygen by NIV or HFNC) | 1.12 (1.06, 1.19) | <0.001 |
|  | | 4 points (intubation and MV) | 2.45 (1.76, 3.43) | <0.001 |
|  | | 5 points (MV with vasopressor use) | 3.83 (2.59, 5.65) | <0.001 |
|  | | 6 points (MV and vasopressor use, dialysis or ECMO) | 12.23 (10.50, 15.32) | <0.001 |
| Diagnosis of ARDS | | | 1.60 (1.38, 2.00) | <0.001 |

OR, odds ratio; CI, confidence interval; COVID-19, Coronavirus disease-2019; CCI, Charlson comorbidity index; WHO, world health organization; NIV. Noninvasive ventilation; HFNC, high flow nasal cannula; MV, mechanical ventilation; ECMO, extracorporeal membrane oxygenation; ARDS, acute respiratory distress syndrome
